# Descriptive review: global spectrum of invasive group B streptococcal disease in nonpregnant adults: epidemiology, risk factors and clinical presentations

**DOI:** 10.1007/s10096-026-05543-z

**Published:** 2026-05-14

**Authors:** Maisa Ali, Almurtada Razok, Abdul Rehman, Walid Al-Wali, Muna Al Maslamani, Hamad Abdel Hadi

**Affiliations:** 1 Communicable Diseases Centre, Division of Infectious diseases, Hamad Medical Corporation, Doha, PO 3050 Qatar; 2 Medical College, Qatar University, Doha, Qatar; 3 Department of Internal Medicine, John H. Stroger Jr. Hospital of Cook County, Chicago, IL 60612 USA; 4 Department of Laboratory Medicine and Pathology, Hamad Medical Corporation, Doha, 3050 PO Qatar

**Keywords:** Group B streptococcus (GBS), Streptococcus agalactiae, Invasive infections

## Abstract

*Streptococcus agalactiae*, or Group B Streptococcus (GBS), is a historical cause of perinatal infections and neonatal sepsis. While routine screening programs in high-income countries have led to a steady decline in neonatal complications, GBS remains a frequent colonizer of the adult gastrointestinal and urogenital tracts. Over recent decades, the incidence of invasive GBS infections in nonpregnant adults has increased substantially, leading to significant morbidity and mortality. This shifting epidemiological landscape is further complicated by the troubling emergence of multidrug-resistant lineages and hypervirulent genomic clones, such as ST283, which demonstrate severe invasive potential and unique zoonotic transmission capabilities. Because collective data on this growing threat remains fragmented, this review synthesizes global literature on invasive adult GBS, encompassing its changing epidemiology, patient risk factors, and its expanding clinical spectrum. The primary objective is to collate updated evidence to raise clinical awareness, support antimicrobial stewardship, and direct future research toward effective preventive measures and strategies.

## Introduction

*Streptococcus agalactiae* or Group B streptococcus (GBS) is a Gram-positive, beta-hemolytic bacterium that colonizes the gastrointestinal and genital tracts of almost one-third of humans. While frequently harmless, it is capable of causing severe invasive diseases, such as fatal meningitis and septicemia, when optimal pathogen, host, and environmental factors align [1, 2]. First isolated in the early 1930 s from livestock affected by bovine mastitis by Rebecca Lancefield, its definitive association with fatal peripartum infections was established in 1938 [3]. The GBS pathogenicity is largely categorized by its outer capsular polysaccharide into 10 different serotypes, though human disease is attributed to the foremost five in the majority of reported cases [1, 4, 5].

During the 1960 s and 1970 s, GBS emerged as a prominent cause of maternal and neonatal sepsis, driving significant morbidity and mortality. In response, high income nations implemented comprehensive preventive measures centered on routine maternal screening and targeted intrapartum prophylactic antimicrobial therapy. These public health efforts successfully mitigated the threat, resulting in a steady epidemiological decline in perinatal GBS infections [6].

However, the epidemiological landscape is shifting. Invasive GBS disease is now increasingly reported in the nonpregnant adults, particularly vulnerable population, presenting a wide and severe clinical spectrum that merits closer evaluation. This rising incidence is particularly notable among older adults across North America, Europe, Asia, and Australia [7]. Because the epidemiology, disease burden, and clinical presentations vary significantly based on host and pathogen characteristics and given the limited published data from certain geographic regions [8], this review aims to collate the available global literature. We collated and synthesized updated evidence regarding evolving epidemiology, affected adult populations, risk factors, clinical spectrum, and preventive measures to better understand and manage this growing threat.

## Methodology

Available publications pertaining to invasive GBS and *Streptococcus agalactiae* infections have been searched in PubMed over the past 25 years between the year 2000–2025. The adopted definition for invasive GBS infection (iGBS) aligns with the widely used combined microbiological and clinical definitions: isolation of *Streptococcus agalactiae*/GBS from sterile sites such as blood, CSF, pleural of peritoneal fluids together with clinical evaluation supportive of infection episodes [9].

The following Booleans search strategies were used for the search: GBS/Group B streptococci OR *Streptococcus agalactiae* AND invasive disease including primary and secondary bacteremia, cellulitis, skin and soft tissues infections (SSTIs), septic arthritis, osteomyelitis, urinary tract infections (UTIs)/genitourinary, pneumonia, meningitis, endocarditis, peritonitis, or intra- abdominal infections (IAIs). Following evaluation, relevant papers and reports published within the outlined period covering adult populations but specifically excluding maternal, perinatal, and neonatal data. Singular case reports were excluded but only included for rare conditions since the prime focus was directed towards comprehensive studies examining one or multiple infection sites. Studies were further classified according to geographical locations, studied years, and examined isolation sites for easier comparative review of Table 1. Summarized data are presented from a wider descriptive perspective outlining the spectrum, epidemiology -including serotypes and genomic details when available -, risk factors as well as various clinical presentations.

**Table 1:** Major studies of GBS invasive disease published between the year of 2000–2025 on adults and nonpregnant women identified by first author and publication year outlying geographical locations, studied years, number of isolates, and affected sites

Study	Country	Studied years	Isolates	BSIs	SSTIs	BJIs	Pneumonia	UTIs	IE	Meningitis	Peritonitis	Reference
Vasikasin 2021	Thailand	2006–2016	282	√								[34]
Ballard 2016	Global	2000–2010	3500	√								[7]
Skoff 2009	USA	1990–2007	19,512	√								[8]
Saad 2018	Argentina	2009–2013	11	√								[38]
Mitsuboshi 2021	Japan	2013–2018	77	√								[39]
Ali,2022	Qatar	2015–2019	196	√	√	√	√	√		√		[29]
Vuillemin 2021	France	2007–2019	1960	√	√	√	√	√	√	√		[27]
Eskandarian 2013	Malaysia	2010–2011	18	√	√	√	√	√	√	√	√	[37]
Park, 2014	Korea	1991–2000	67	√	√	√					√	[67]
Morozumi 2016	Japan.	2010–2013	443	√	√	√	√					[18]
Edwards 2005	USA	2003	1328	√	√	√	√	√	√			[36]
Phares 2008	USA	1999–2005	14,573	√	√	√	√	√	√	√	√	[45]
Jump 2019	USA	2008–2017	5497	√	√	√	√				√	[63]
Collin 2019	Global	2000–2017	1890		√							[40]
Smith 2015	USA	1995–2012	8300			√						[41]
Kernéis 2017	France	2004–2014	163			√						[42]
Ong 2018	Singapore	2010–2015	54			√						[43]
Louthreno 2014	Thailand.	2008–2010	244			√						[44]
Skolnik 2017	Canada	1978–2013	318				√					[47]
Ulett 2009	USA	2007–2008	16,000					√				[52]
Mongilardi 2022	USA	2008–2017	21,000					√				[51]
Sambola 2002	Spain	1962–1998	31						√			[54]
Oravec 2022	Canada	2000–2018	28						√			[56]
Phoompoung 2021	Thailand	2013–2017	15							√		[58]
Trollfors 2022	Sweden	2003–2016	16								√	[68]

*BSIs* blood stream infections, *SSTIs* skin and soft tissues infections, *BJIs* bone and Joints infections, *UTIs* urinary tract infections, *IE* infective endocarditis

### Spectrum

Despite being mainly colonizer at the gastrointestinal and genital tract of humans, invasive GBS disease has been associated with wide disease spectrum in a variety of organs including but not limited to genital, skin, and soft tissues (SSTs), bone and joints infections (BJIs), urinary tract, isolated bloodstream infections (BSIs), respiratory, and cardiovascular infections such as pneumonia and endocarditis as well as central nervous infections such as ventriculitis and meningitis [10] Table 1. Despite not being a prominent disease entity for many healthcare and public health professionals, the spectrum of GBS associated disease has been compared to the well-recognized pneumococcal disease with experts calling for wider awareness and preventive measures [11].

### Global epidemiology

**Fig. 1:**
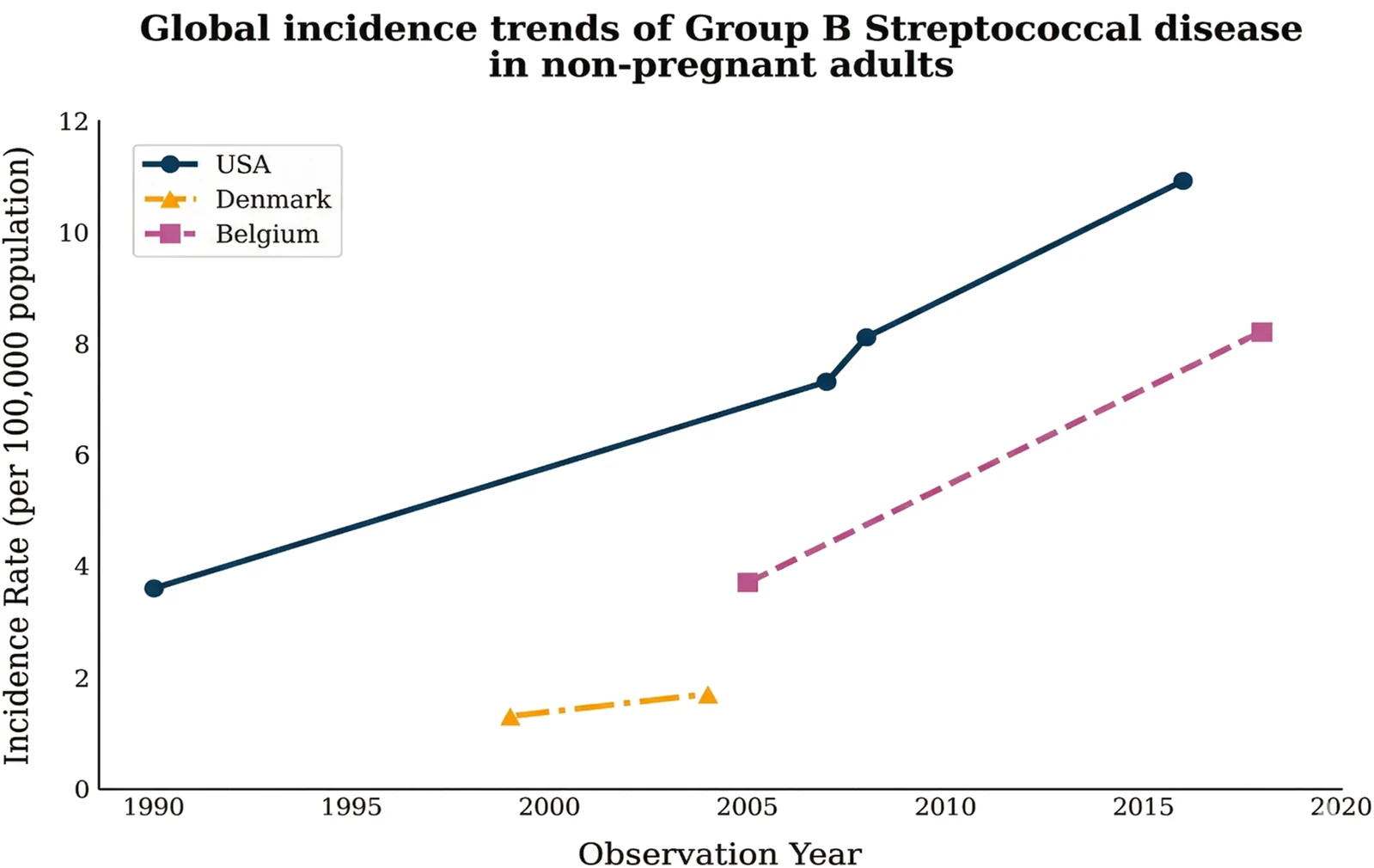
Examples of the global incidence trends of Group B streptococcal disease in nonpregnant adults based on surveillance studies on corresponding years. The graph trajectory projects escalating trends in USA, Denmark and Belgium between 1990–2020

In adult populations, the incidence of GBS varies by geographic location; however, it is safe to report that there is a global accelerating trend of invasive GBS infections in the last two decades [12]. From an estimated global pool of data analyzed in systematic review and meta-analysis from major continents, the overall global incidence of invasive GBS disease in nonpregnant adults was estimated at 2.86 per 100.000 population being higher in Western when compared to Asian and African countries but with clear rising incidence in older age groups. For example, incidence more than doubles (9.13 to 19.40 per 100,000 population) when comparing ages between ≥ 50 and ≥ 65 ranges with overall case fatality rates of about 10% [13]. Regionally, longitudinal epidemiological studies from USA between 2008 and 2016 representing almost 10% of the population with over 21,000 invasive GBS episodes, estimated incidence of invasive GBS infections in nonpregnant adults in 2008 as 8.1 cases per 100,000 population, which increased to 10.9 by the year 2016. Similarly in England between 2015 and 2016, the annual incidence in adults was estimated at 3.48 per 100,000 population being higher in the elderly population, however unlike the USA, it was higher in females of childbearing age [9]. Furthermore, a study performed in Belgium between 2005 and 2018, also showed an upward trend in the annual incidence of invasive GBS infections in nonpregnant adults, with an increase from 3.7 to 8.2 cases per 100,000 population Fig. 1 [14].

### Epidemiology of serotypes and clonal complexes

**Fig. 2:**
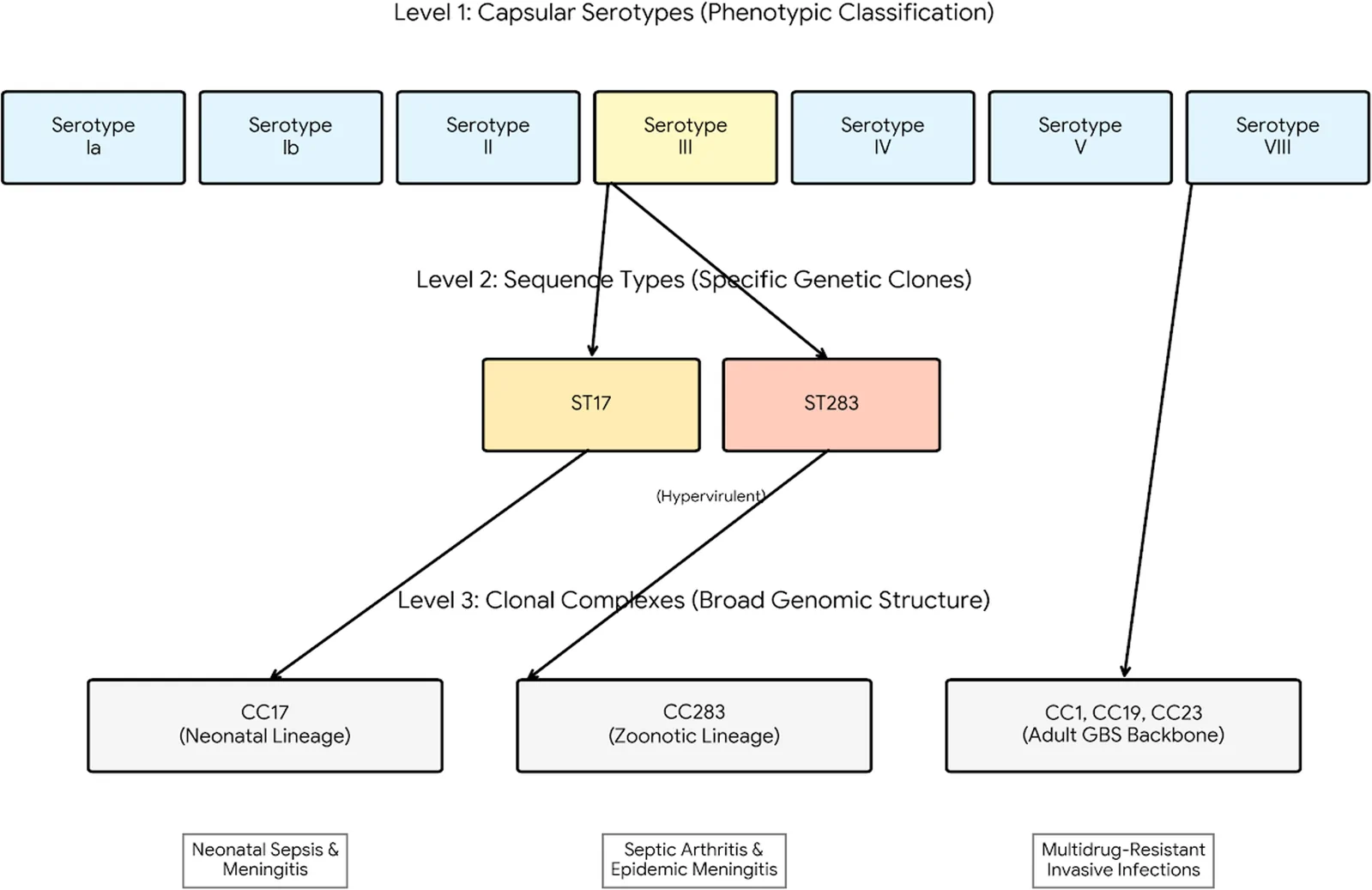
Genomic hierarchy explaining the relationship between serotypes, sequence types and clonal complexes with examples of invasive *Streptococcus agalactiae* classification. *GBS* group B streptococcus , *ST* sequence type , *CC* clonal complex

To understand the pathogenicity and global spread of *Streptococcus agalactiae*, it is essential to distinguish between its three primary molecular classification systems: capsular serotypes, sequence types (STs), and clonal complexes (CCs). The capsular serotype represents the most traditional classification, defined by the chemical structure of the outer polysaccharide coat; while 10 serotypes exist, human disease is primarily driven by the leading five serotypes I - V. Nevertheless, the physical coat does not always reflect the bacterium’s deeper genetic behavior. Sequence Typing (ST), determined via Multi-Locus Sequence Typing (MLST) of specific genome sequencing loci, identifies specific genetic clones through analyzing variations in seven internal housekeeping genes. Additionally, closely related sequence types are further grouped into Clonal Complexes (CCs), that group close relatedness to examine broad ancestral lineages. Hence, serotypes and STs represent two separate entities that should not be interchanged when reviewing the pathogen genetic epidemiology. This distinction is critical because while serotypes are the focus for vaccine development, specific genetic clones—such as the hypervirulent ST283 or ST 17 — exhibit unique transmission and increased invasiveness that might not be captured by serotyping alone [13, 15] Fig. 2 and Table 2.

**Table 2:** Summary of methods for the classifications of GBS disease

Classification	Method	Scope	Key findings from clinical reports	Referenced studies
Serotypes	Capsular Polysaccharide	Physical surface	Predominant Types: Ia, Ib, II, III, and V cause the majority of human diseases. Serotype Ia is frequently dominant among nonpregnant adults, while Serotype V is a leading cause of UTIs. No strong evidence for gender or ethnicities differences between serotypes	[8, 17, 52, 69]
Sequence Types (ST)	Muti-locus sequence typing (MLST) using conserved housekeeping genes	Specific clone	ST283: A hypervirulent clone associated with serotype 3. It is responsible for foodborne outbreaks linked to raw fish and causes severe septic arthritis and meningitis in healthy adults.	[23, 24]
Clonal Complex (CC)	Grouped STs	Broad Lineage	Predominant Lineages: CC1, CC19, and CC23 drive adult GBS epidemiology. These lineages are often associated with multidrug resistance to macrolides and tetracyclines	[70, 71]

Global systematic review of invasive GBS disease amongst nonpregnant adults focusing on reported serotypes and clonal complexes (CC), identified serotypes V and CC 1 predominance, while serotypes III, Ia, Ib, II and IV complete the foremost reported serotypes [13]. Although there were reports linking gender and ethnicity with certain serotypes, there was no strong consensus to direct reliable conclusions [16, 17]. Notably, in a large US study, incidence was higher in males, mainly of black ethnicity and patients > 80 years of age while serotypes 1,2,3 and 5 represented almost 90% of isolates [17]. In contrast, in a large nationwide study from Germany encompassing almost 1000 invasive GBS isolates (the majority were BSIs from adult populations > 65 years) serotype V predominates followed by serotype 1–2 mainly without observed gender differences [16]. Conversely, serotype 1a of adult populations was identified in a study from Portugal while a study of 443 patients from Japan recorded non-focused bacteremia as the main presenting picture in adults with predominance of serogroups 1b, followed by 5, 3, 4 and being least for 1a [18]. More plausibly, regional and isolation sites might provide serotype differential bases Table 3. For example, in North America serotypes V is the most reported in adult populations as opposed to serotype III, which is the most reported in Europe while bacteremia study from Korea outlined serotype 8 as the main predominant [5, 13, 19].

**Table 3:** GBS serotypes, sequence types and clonal complexes with reported clinical spectrum

Classification	Dominant Clinical Presentation	Supporting evidence
Serotype Ia	Non-focused bacteraemia/invasive disease	Identified as the dominant serotype in nonpregnant adults in the USA and Portugal.
Serotype III	Meningitis and Bone/Joint infections	Associated with adult outbreaks (ST283).
Serotype V	Urinary Tract Infections (UTIs)	Consistently recognized as a leading cause of GBS-associated UTIs.
Serotype 8	Bacteraemia	Predominat in South Korea
ST17 (CC17)	Neonatal Sepsis and Late-Onset Meningitis	The primary hypervirulent clone driving neonatal complications
ST283 (CC283)	Septic arthritis and epidemic meningitis	Specifically linked to severe outbreaks in healthy adults following raw fish consumption.
CC1, CC19, CC23	Multidrug-Resistant Invasive infections	These broad lineages drive the overall adult population structure and are frequently associated with AMR profiles

Distinctively, recent advances in whole-genome sequencing (WGS) have profoundly reshaped our understanding of the genomic epidemiology of invasive GBS in adults, highlighting the troubling emergence and clonal expansion of multidrug-resistant lineages. The adult GBS population structure is predominantly driven by distinct clonal complexes (CCs), notably CC1, CC19, and CC23, which frequently exhibit lineage-specific virulence factors and antimicrobial resistance (AMR) profiles. Of particular concern is the escalating prevalence of macrolide and tetracycline resistance—mediated by genes such as *ermB*, *mefA*, and *tetM*—which are frequently mobilized by integrative and conjugative elements (ICEs) across diverse capsular serotypes [20, 21]. Furthermore, high-resolution genomic surveillance has identified novel point mutations in penicillin-binding proteins (PBPs) that confer reduced susceptibility to first-line beta-lactam therapies. This genomic plasticity, is compounded by the acquisition of prophages and other mobile genetic elements that enhance niche adaptation, tissue invasion, and immune evasion. The recent outbreak of the highly virulent sequence type (ST)283, which demonstrated a unique zoonotic transmission capabilities and severe invasive potential, further underscores the dynamic evolutionary nature of the GBS genome [22].

While neonatal invasive disease is frequently driven by the ST17 clone—a hypervirulent lineage typically associated with capsular serotype III and vertical transmission —the epidemiology of adult disease has been reshaped by the emergence of ST283. ST283 is genetically distinct from the common ancestral lineages that dominate adult GBS, such as CC1, CC19, and CC23, and is often classified within its own unique lineage, CC283. A defining characteristic of ST283 is its specific pathogenic traits, including enhanced gastrointestinal invasiveness and cross-species infectivity, which allow it to bypass host defenses in relatively healthy individuals. Unlike ST17, which primarily causes neonatal sepsis and meningitis, ST283 represents a significant foodborne zoonotic threat linked to raw freshwater fish, driving outbreaks of invasive septic arthritis and epidemic meningitis in adults. This distinction highlights the dynamic evolutionary nature of the GBS genome and the critical need for molecular surveillance to track clones with high-resolution sequence typing rather than relying on capsular serotyping alone [23, 24]. Consequently, integrating routine genomic epidemiology is paramount not only for tracking the global spread of these multidrug-resistant bacteria but also for guiding the formulation of future polyvalent vaccines and refining targeted antimicrobial stewardship for vulnerable adult populations [22, 25].

### Patient characteristics and risk factors

For invasive GBS disease in nonpregnant adult populations, old age (> 65) has been identified as an independent risk factor from multiple studies [9, 26, 27]. Risks of invasive disease more than quadruple when comparing patients > 65 year of age to young adults[13]. Regarding gender differences, because of anatomical colonization with GBS is more common in females than males, some studies on the invasive disease captured the plausible association of females’ preponderance while others pointed towards the opposite particularly amongst elderly population [9]. In a large observational study from USA of over 5500 episodes of invasive GBS disease conducted between 2008 and 2017, extremes of BMIs (BMI < 18 and > 40) in addition to poor glycemic control (HBA1 C > 9.5) were associated with a fourfold risk increase [28]. The positive association with diabetes mellitus (DM) has been firmly observed in other similar studies [9, 14, 29]. In addition to old age and poorly controlled DM, invasive GBS disease also tends to affect patients with underlying chronic comorbidities since surveillance studies from Europe highlighted prevalence of underlying medical conditions such as cardiovascular, pulmonary, and chronic kidney diseases in most of the affected population [9, 14]. Similarly, a study from Korea highlighted chronic medical conditions in over 90% of reported cases [30]. Additionally, a study from Denmark pointed towards excessive alcohol and underlying neoplastic diseases [26].This positive correlation with comorbidities has been cemented following the analysis of global data extracted at systemic reviews and metanalysis [13].

## Zoonotic transmission and the emergence of clone ST283

Of note, it is intriguing to notice that GBS is a recognized zoonotic pathogen for farmed freshwater fishes, where in Singapore in late 2015, a large foodborne GBS associated disease outbreak was eventually traced to eating raw fishes [31]. The Singapore outbreak fundamentally shifted the epidemiological understanding of GBS, establishing it as a severe foodborne zoonotic pathogen. This unprecedented epidemic affected more than 400 patients and was definitively traced to the consumption of raw freshwater fishes contaminated with the hypervirulent sequence type 283 (ST283) clone. Genomic and epidemiological analyses have shown that ST283, which falls under capsular serotype 3, possesses unique pathogenic traits that facilitate cross-species infectivity [31]. Notably, unlike classic opportunistic GBS strains that predominantly afflict the elderly or those with chronic comorbidities, ST283 has demonstrated an alarming capacity to cause invasive disease in relatively healthy adults. The clinical manifestations of this specific foodborne transmission are exceptionally severe, with the ST283 clone being a primary driver of invasive septic arthritis and epidemic meningitis. The ability of this virulent clone to bypass normal host defences and cause profound systemic infections via the gastrointestinal route underscores a critical intersection between regional aquaculture practices, culinary habits, and public health. Recognizing the zoonotic mechanisms of ST283 is therefore essential for developing targeted food safety guidelines and active molecular surveillance to prevent future cross-species outbreaks [32].

### Clinical presentations and disease spectrum

#### Bloodstream infection

A multinational population based study conducted in nine global high-income regions in Australia, Europe and North America between 2000 and 2010 covering almost 3500 cases of GBS BSIs from all age groups, demonstrated an overall rising trend particularly evident in the elderly population of almost double rates (5.6–10.6 per 100.000 patients years) [7].Similarly, although the incidence of GBS bacteremia in pregnant women and neonates has been declining in the USA, the rate has been increasing in nonpregnant adults [8, 33].That was evident when a bacterial core surveillance study conducted between 1990 and 2007 in the USA including 10 states, detected 19,512 cases of invasive GBS infections in the adult populations older than 18 years of age, and demonstrated a doubling in the rate of incidence from 3.6 to 7.3 cases per 100,000 population [8]. Additionally, a study from Thailand of 282 episodes of GBS BSIs reported an increased trend, observable seasonal variations with an overall 30-day mortality of 16% [34].

A striking feature of invasive GBS BSIs in the adult populations is that bacteremia without a clear focus is a leading manifestation of the disease particularly in elderly population [27, 34–36]. However, GBS BSIs can occur secondary to a primary focus of infection especially SSTIs and pneumonia, or concurrently with other bacteria in the form of polymicrobial bacteremia [5]. Intriguingly, *Staphylococcus aureus* was the most common associated pathogen from polymicrobial BSIs in some studies [37]. Commonly reported presentations include constitutional symptoms such as fever, chills, and rigors albeit fever can be absent in some reported cohort [29]. Associated risk factors are similar to other invasive GBS infections which include advanced age, diabetes mellitus, obesity, immune suppression, renal and liver diseases [38]. Additionally, in the far East, eating raw fish and liver cirrhosis has been identified as an independent risk factor with significant morbidity and mortality [24]. Since GBS are typically sensitive to penicillin, they remain the prime recommended antimicrobials for BSIs with infrequent resistance rates while cephalosporins and glycopeptides are the recommended alternative therapy [5]. Despite highlighted escalating resistance rates to macrolides, a Japanese study of 77 cases of GBS BSIs found that phenotypic quinolones or macrolide resistance are unlikely to contribute to poor outcomes[39].

#### Skin and soft tissue infections (SSTIs)

A systematic review performed by Collin et al. in 2019 covering sixty-seven countries estimated the overall prevalence of GBS in SSTI isolates as 1.89%. For surgical site infections (SSIs), GBS was isolated in 0.73% of all types of surgery, with the highest prevalence observed following caesarean sections ranging between 2.92% and 9.69% [40]. Furthermore, a retrospective population-based surveillance performed in the USA estimated the incidence of GBS cellulitis in 2012 as 3.8 cases per 100,000 population, increasing from 1.6 in 1995. Most cases were observed in males, patients older than 80 years old, and Hispanics while diabetes mellitus was the most frequent comorbid condition [41].

The spectrum of the GBS disease encompasses a variety of SSTIs that include both superficial and deep-seated infections ranging from mild to life-threatening conditions. Cellulitis, paronychia, skin abscesses, diabetic foot and pressure ulcer infections were reported, as well as pyogenic myositis and necrotizing fasciitis [10]. Immunocompromising conditions such as diabetes mellitus and advanced age are among the most prominent risk factors [35].Other pertinent risk factors include immobility, chronic medical conditions such as congestive heart failure, chronic liver diseases, immune suppression such as neoplastic and chronic kidney diseases [36]. A combination of physical and immunochemical factors contributed to the increased risk of GBS SSTIs, especially in the elderly population. These included disruption of normal anatomical barriers, increased burden of colonization, medical instrumentation, altered host immune response such as reduced innate and adaptive immune reactivity, and delays in diagnoses [36].

#### Bone and joint infections (BJIs)

A population-based surveillance performed in USA showed an increase in the incidence of GBS septic arthritis and osteomyelitis from 5.8 to 8.3 cases per 100,000 of population from 1995 to 2012.Studied risk factor outlined that most cases had at least one underlying medical comorbidity. The most prevalent condition was diabetes mellitus followed by obesity, smoking, renal disease, and malignancy [41]. Additionally, a retrospective analysis performed by Kernéis et al. in France, from January 2004 to December 2014 showed that most cases occurred in patients more than 50 years of age who had at least one underlying medical condition, with the most common being solid malignancies, followed by diabetes mellitus with prevalence of 21% and 20%, respectively while the most frequent affected joints were the knee, followed by the hip at 30% and 27%, respectively [42].

Exploring pathophysiology, GBS BJIs may occur in both native and prosthetic joints due to hematogenous spread, direct extension from skin and soft tissue infections, or disruption of overlying skin such as pressure ulcers. Furthermore, GBS serotype 3 and clonal ST283 have been associated with invasive GBS septic arthritis in relatively healthy adults’ points towards potential virulence factors [43]. Fever and arthralgia were among the most common presenting symptoms, and bacteremia was associated with over 80% of cases in one study. It seems that biofilm production plays a significant role in the pathogenesis of GBS and BJIs [44].

#### Pneumonia

A population-based surveillance study performed in the USA between 1999 and 2005 including 10 states of almost 15,000 patients including 6087 adults, estimated the prevalence of pneumonia at 11% of all GBS invasive infections in adults, coming third following bacteremia without a focus and SSTIs matched by a similar study from Japan demonstrating prevalence at 9% and similar frequency [45]. Additionally, a review of almost 2000 cases of GBS pneumonia demonstrated risk factors of old age with a mean age of 70 years and multiple comorbidities including pulmonary, heart and renal diseases. Curiously, in 36% of cases GBS pneumonia was associated with bloodstream infection pointing towards significant virulence with overall mortality of 15% [28].

While GBS historically was known to be associated with neonatal pneumonia, its implication with lower respiratory tract infections in the adult nonpregnant population became more recognized during the 1980s. Initially described as a cause of nosocomial pneumonia with high mortality, GBS was later found to be associated with pneumonia mainly in the elderly population who had predisposing conditions such as central nervous system dysfunction and swallowing difficulties pointing towards probable aspiration route from adjacent gastrointestinal tract [36]. That might explain that GBS pneumonia requires certain host factors since it is an uncommon cause of adults’ community acquired pneumonia [46]. Comparably, despite patients with cystic fibrosis (CF) having chronic lung disease with frequently isolated GBS from the sputum, review of multiple cohorts demonstrated it is an uncommon cause of pneumonia [47].

#### Urinary tract infections (UTIs)

GBS is one of the organisms that colonize the lower Genito-urinary tract with a prevalence rate of almost one third of affected population[5]. That might explain the shift from colonization towards invasive disease such as related UTIs when pathogen, host and local environmental factors become optimal. Besides being one of the reported pathogens causing UTIs and asymptomatic bacteriuria in the pregnant population, GBS has been associated with a wide range of urinary disease spectrum in the adult nonpregnant populations including asymptomatic bacteriuria, cystitis, pyelonephritis, prostatitis, urethritis and urosepsis [48]. Out of invasive GBS disease in the adult nonpregnant population, a population-based study revealed GBS associated UTIs account for 14% of cases however, interestingly, that rises to 39.4% in adults over the age of 70 [35, 48]. Over the last three decades, the incidence of GBS UTIs accounted for almost 2% of all UTIs, and 4.5% in patients with type II diabetes mellitus [49]. For example, in a study of almost 35,000 urine samples with suspected UTIs, GBS was isolated in 1.1% of cases with predominance of serotype 5 while a large study of 700 confirmed cases of GBS UTIs from Argentina, identified serotype 1a and 3 [50]. Moreover, in a large study between 2008 and 2017 encompassing around 21,000 episodes of GBS bacteriuria, UTIs were actively considered and treated in only 15% of cases being associated with an observable higher 30 day mortality. Presenting symptoms were similar to UTIs caused by other organisms and included constitutional symptoms such as fever, chillis, flank pains, dysuria, frequency and urgency [51]. Despite advances in microbiological techniques, the clinical distinction between GBS asymptomatic bacteriuria and true invasive infection remains notoriously elusive, particularly as quantitative culture methods are often indiscriminative and fail to provide a definitive diagnosis. This diagnostic ambiguity poses a substantial clinical challenge, frequently leading to the overdiagnosis of UTIs and the subsequent inappropriate prescribing of antimicrobial therapy. In the context of hospital-acquired infections, the overtreatment of GBS asymptomatic bacteriuria acts as a significant driver of antimicrobial resistance, placing vulnerable adult populations at risk for collateral complications as well as the emergence of multidrug-resistant organisms. Furthermore, the misclassification of simple colonization as an active infection triggers a cascade of unnecessary diagnostic interventions, inappropriate consultations, and prolonged hospital stays, which substantially increase the economic costs and overall healthcare burden. Therefore, implementing rigorous, symptom-based diagnostic criteria and promoting active antimicrobial stewardship are paramount to mitigating the unintended clinical and economic consequences of treating asymptomatic GBS colonization [52]. Additionally, in a study of almost 16,000 of confirmed GBS urinary samples, main risk factors for UTIs included age > 40 and diabetes mellitus while other identified risk factors were similar to other causes of UTIs which include indwelling urinary catheters, obstructive uropathy and prostatic hypertrophy [52].

#### Infective endocarditis

Initially identified as a cause of infective endocarditis (IE) in pregnant and post-partum patients in the 1930s, GBS’s association with endocarditis in nonpregnant adult populations surfaced during the1960s [53]. Generally, GBS related cardiovascular disease including IE is rare but has significant morbidity and mortality as well as tendency of recurrence [17]. As an elaboration, in a Spanish study spanning almost 25 years covering almost 1800 episodes of IE, only 31 (1.7%) of cases of GBS related endocarditis were identified albeit with high rates of secondary complications and high mortality at 47% [54]. Similarly in a study of over 300 episodes of IEs, isolates of GBS cases were only 3%, the majority of which developed complications [55]. Furthermore, in a nested case control study over 18 year period conducted in Canada encompassing 520 episodes of GBS BSIs, only 5.4% of cases fulfilled Dukes criteria for definite or probable categories for IE however the in-hospital mortality rate was high at 29%. Apart from these surveillance studies, most of the available literature describes case reports and series rather than substantial data. From cohort and reported cases studies, described risk factors, complications and outcomes, highlighted that both native and prosthetic valves are affected including mitral, tricuspid, and aortic valves while key risk factors include old age, previous history of rheumatic heart disease, drug abuse, GBS BSIs as well as debilitating and neoplastic diseases [56].

#### Meningitis

Historically GBS meningitis has been widely described as neonates causing significant morbidity, and mortality [57]. Inversely, adult GBS meningitis is rare and mostly encountered in patients with certain risk factors. In a systematic review of the literature between 1975 and 2018, only 141 cases were identified in adults citing immunocompromised state, CSF leakages, and infective endocarditis as risk factors. Additionally, in study of 224 episodes of GBS disease between 2013 and 2017, meningitis was the least common manifestation at 7% [58].Described presenting symptoms were similar to those of bacterial meningitis which included fever, headache, neck stiffness, and altered mental status. Additionally, complications were also similar which included seizures, sepsis, and transient or permanent neurological deficits but with high mortality rates at 31% being significantly higher than those reported for *Streptococcus pneumoniae* [59]. Furthermore, described risk factors include, old age, immune suppression, diabetes, alcoholism, presence of distant focal infections such as infective endocarditis while advanced age, and complications at presentation were the strongest risk factors for poor outcomes [60]. Intriguingly, from a community outbreak of foodborne disease, significant epidemic GBS meningitis cases were caused by serotype 3 which was traced to eating raw fish in Singapore [61].

#### Peritonitis

Generally, streptococci are rare causes of spontaneous or secondary bacterial peritonitis since in a study over 20 years only 32 patients were diagnosed as primary Group A *Streptococcus* (GAS) spontaneous peritonitis while in a study over 6 years, 84 streptococcal pathogens were implicated in dominated by viridans with GBS isolated is 8.4% of cases [62]. Hence, there are scarce data about GBS peritonitis in the literature, for example from a large study in USA of 5497 disease episodes, GBS was only isolated from peritoneal fluid in only 138 of cases (2.5%) while a study of 1244 episodes of GBS isolated from sterile sites, only16 (1.3%) were isolated from peritoneal fluids [63]. Nevertheless, GBS can cause rare secondary and less frequent primary peritonitis since there are documented reported cases being associated with highest mortality when compared to other isolated sites [63]. In patients with continuous ambulatory peritoneal dialysis (CAPD), it seems most likely that the infection originates from colonized patients or their partners since some reported cases isolated identical clones from relevant sites [64]. Of note, other reported risk factors for spontaneous GBS peritonitis include invasive gynecological procedures in colonized patients and biological treatment such as Janus Kinase inhibitor, ruxolitinib resulting in documented disease manifestations [65].

#### Control and prevention

Following the control of GBS related disease in the perinatal period, comprehensive prevention and control measures should be shifted to control the disease in the adult non pregnant populations. The scale of shifting epidemiology points towards substantial challenges of invasive GBS disease affecting adults and nonpregnant women particularly the elderly with significant morbidity and mortality [11, 13]. Since GBS has distinct polysaccharides characterizing their different serotypes with limited predominant ones causing human disease, it is plausible to assume the development of a polyvalent vaccine will aid the control of the disease specially for at risk populations such as the elderly and patients with chronic medical conditions such as diabetes or immune dysfunction[15]. Fortunately, there are a GBS vaccines at advanced stages of development supported by the WHO as a public health priority to combat the escalating and serious disease [66].

## Conclusion

Invasive GBS related disease in adults and nonpregnant women is an emerging pathogen in multiple geographic locations associated with significant morbidity and mortality specially in distinct population. From multiple epidemiological studies, it is evident the disease is associated with upwards trends particularly for the elderly and vulnerable population such as patients with diabetes and chronic comorbidities where the disease can cause substantial health challenges presenting as wide disease spectrum spanning all systemic organs. The presented review tried to address gaps in disease knowledge to raise awareness for the medical community toward optimal management as well as open gates for future research and development including control and prevention strategies.

## Data Availability

We used readily available data from literature review available in PubMED.

## Declarations

### Use of artificial intelligence

During the preparation of this work the authors used Google Gemini in order to produce illustrative figure out of generated data. After using this tool, the authors reviewed and edited the content as needed and take full responsibility for the content of the published article.
